# Prevalence and prevention of suicidal ideation among asylum seekers in a high-risk urban post-displacement setting

**DOI:** 10.1017/S2045796022000579

**Published:** 2022-10-17

**Authors:** A. Aizik-Reebs, K. Yuval, Y. Beyene Kesete, I. Lurie, A. Bernstein

**Affiliations:** 1 University of Haifa, Haifa, Israel; 2 Shalvata Mental Health Center, Hod Hasharon, Israel; 3 Department of Psychiatry, Sackler School of Medicine, Tel Aviv University, Tel Aviv, Israel

**Keywords:** Asylum seekers, depression, forced displacement, mindfulness, refugees, suicidal ideation, suicidality, trauma

## Abstract

**Aims:**

Among asylum seekers in a high-risk unstable post-displacement context, we aimed to investigate the prevalence of and risk for suicidal ideation (study 1), and then to test whether and how Mindfulness-Based Trauma Recovery for Refugees (MBTR-R) may prevent or treat suicidal ideation (study 2).

**Methods:**

Study 1 was conducted among a community sample of *N* = 355 (31.8% female) East African asylum seekers in a high-risk urban post-displacement setting in the Middle East (Israel). Study 2 was a secondary analysis of a randomised waitlist-control trial of MBTR-R among 158 asylum-seekers (46.2% female) from the same community and post-displacement setting.

**Results:**

Prevalence of suicidal ideation was elevated (31%). Post-migration living difficulties, as well as posttraumatic stress, depression, anxiety and their multi-morbidity were strongly associated with suicidal ideation severity. Likewise, depression and multi-morbidity prospectively predicted the onset of suicidal ideation. Relative to its incidence among waitlist-control (23.1%), MBTR-R prevented the onset of suicidal ideation at post-intervention assessment (15.6%) and 5-week follow-up (9.8%). Preventive effects of MBTR-R on suicidal ideation were mediated by reduced posttraumatic stress, depression, anxiety and their multi-morbidity. MBTR-R did not therapeutically reduce current suicidal ideation present at the beginning of the intervention.

**Conclusions:**

Findings warn of a public health crisis of suicidality among forcibly displaced people in high-risk post-displacement settings. Although preliminary, novel randomised waitlist-control evidence for preventive effects of MBTR-R for suicidal ideation is promising. Together, findings indicate the need for scientific, applied and policy attention to mental health post-displacement in order to prevent suicide among forcibly displaced people.

## Introduction

We are in the midst of a global crisis of forced displacement (Patel *et al*., 2018). Worldwide, tens of millions of refugees and asylum seekers have been forcibly displaced by war, violence and persecution (UNHCR 2020). The trauma and stress experienced before, during and following forced migration contribute to high rates of stress- and trauma-related mental health problems (Priebe *et al*., 2016). We focus here on one particularly destructive consequence of this crisis – suicidality and, specifically, suicidal ideation, post-displacement among adults (WHO 2014; Colucci *et al*., 2017; Ventevogel *et al*., 2019).

A limited number of existing studies have focused on suicidality among adult refugee populations who have the relative fortune of residing in stable resettlement contexts post-displacement, most typically in high-income countries (Vijayakumar, 2016; Colucci *et al*., 2017). In stable re-settlement post-displacement contexts, point- and lifetime-prevalence of suicidal ideation among resettled refugees (3–18.7%) (Jankovic *et al*., 2013; Ao *et al*., 2016; Nickerson *et al*., 2019) tend to be similar or slightly elevated relative to Western Educated Industrialised Rich Democratic (WEIRD) populations and migrants (3–15.9% and 3.4–16.1%, respectively) (Nock *et al*., 2008; Amiri, 2020). Similarly, population-registry studies have documented 90:100 000 suicide attempts and 11: 100 000 suicides per annum among resettled refugees in Sweden relative to 105: 100 000 suicide attempts and 20: 100 000 suicides among Swedish host population (Hollander *et al*., 2020; Amin *et al*., 2021).

In contrast, very little is known about suicidality among asylum seekers in unstable, often temporary, post-displacement or humanitarian settings, without recognised residential status, and most typically in low- or middle-income countries. This is critical, because it is in these higher-risk post-displacement contexts where ~85% of forcibly displaced people (FDP) seeking asylum currently reside (Guterres and Spiegel, 2012; UNHCR 2020). It is this increasingly common post-displacement context – characterised by a multitude of post-migration living stressors, barriers to trauma recovery and thereby elevated risk for stress- and trauma-related mental health problems – where suicidal ideation and related forms of suicidality among refugees and asylum seekers may be most likely elevated (Vijayakumar, 2016; Nickerson *et al*., 2019). Thus, epidemiologic study of suicidal ideation among asylum seekers in these high-risk post-displacement settings is urgently needed (Aichberger, 2014; Colucci *et al*., 2017; Ventevogel *et al*., 2019; Haroz *et al*., 2020).

Consistent with these concerns, initial studies in convenience and community samples of asylum seekers without formal visa status, even in stable, high-income post-migration re-settlement settings, have documented worrying levels of suicidal ideation. Point-prevalence suicidal ideation of 33.9% was observed among refugees in Swedish asylum accommodations and 39.5% among refugees in Australia (Leiler *et al*., 2019; Nickerson *et al*., 2019). Likewise, studies of convenience samples of FDPs residing in refugee camps and among internally displaced people are also concerning – point-prevalence of suicidal ideation of 27.3–29.2% was observed in refugee camps in Nigeria and Uganda, 32.8% among Afghan refugee mothers in a refugee camp in Pakistan and a startling 62% among Rohingya mothers in refugee camps in Bangladesh (Rahman and Hafeez, 2003; Ssenyonga *et al*., 2013; Akinyemi *et al*., 2015; Tay *et al*., 2019).

A related set of studies in clinical samples of refugees, even in stable post-migration settings, indicate that point-prevalence rates of suicidal ideation are markedly elevated and range from 27.8% in asylum seekers in a psychiatric clinic in Switzerland, 29.2% among treatment-seeking refugee survivors of torture in the USA, to 54.1% among refugees in an outpatient clinic in Germany (Lerner *et al*., 2016; Belz *et al*., 2017; Premand *et al*., 2018). These select clinical sample findings are important in that they illustrate the theorised role of stress- and trauma-related mental health problems post-displacement for suicidal ideation (Franklin *et al*., 2017).

Despite the scale and urgency of this public health crisis, only a small number of intervention studies to prevent incidence or reduce pre-existing suicidal ideation among FDPs have been tested to date. A recent systematic review documented that interventions have demonstrated no, insufficient or only partial evidence of efficacy with respect to reduction or prevention of suicidality among FDPs (Vijayakumar, 2016; Singla *et al*., 2017; Haroz *et al*., 2020). Furthermore, although there are a number of additional, stress- and trauma-related mental health interventions for refugees and asylum-seekers, their potential therapeutic efficacy for suicidality has yet to be tested (Barbui *et al*., 2020; Tol *et al*., 2020).

We focus on one such promising approach – mindfulness-based interventions (MBI) (Tol *et al*., 2020; Aizik-Reebs *et al*., 2021) and their potential to prevent the onset of, or treat and thereby reduce pre-existing levels of, suicidality among forcibly displaced populations. First, MBIs or interventions with elements of mindfulness practices show promising therapeutic efficacy for stress- and trauma-related mental health problems among refugees and asylum seekers (Tol *et al*., 2020; Aizik-Reebs *et al*., 2021). A MBI, specifically developed to promote mental health among diverse forcibly displaced populations, Mindfulness-Based Trauma Recovery for Refugees (MBTR-R), has demonstrated randomised waitlist-controlled evidence of its efficacy to significantly improve rates and symptom severity of PTSD, depression, anxiety and their multi-morbidity among traumatised East African asylum seekers (Aizik-Reebs *et al*., 2021). Second, Mindfulness-Based Cognitive Therapy in WEIRD populations has demonstrated robust depression relapse effects important to prevention of suicidality (Mann *et al*., 2005; Piet and Hougaard, 2011), dissociation between depressive symptoms and suicidal cognitions (Barnhofer *et al*., 2015), and reduced suicidal ideation among patients with residual depressive symptoms (Forkmann *et al*., 2014). Third, MBIs may be well-suited to some of the implementation challenges facing refugee mental health intervention and suicide prevention efforts in unstable post-migration settings (Patel *et al*., 2018; UNHCR 2020; Aizik-Reebs *et al*., 2021). Yet, despite the promising initial evidence of safety, efficacy and feasibility of MBIs, to the best of our knowledge, no study to date has tested the effects of a MBI to prevent incidence or reduce suicidal ideation among FDPs broadly, let alone in unstable post-migration settings specifically where risk for suicidality is most likely elevated.

## Aims

In study 1, we estimated the point-prevalence and severity of, as well as candidate risk markers (e.g. post-migration stressors, trauma history exposure) and factors (e.g. trauma- and stress-related mental health symptoms) for, suicidal ideation among an East African community sample of asylum seekers without recognised residential status, residing in an unstable, urban, post-displacement setting in the Middle East (Israel) (*N* = 355, 31.8% female). In study 2, we, first, tested the prospective stability of suicidal ideation among those with and without current suicidal ideation; and whether candidate risk markers and factors at baseline prospectively predict suicidal ideation incidence/onset among a community sample of Eritrean asylum seekers (*N* = 158, 46% female). Second, we tested whether, relative to a wait-list control, a mindfulness- and compassion-based intervention tailored to FDPs (Aizik-Reebs *et al*., 2021) could help *prevent* the incidence or onset of suicidal ideation among asylum seekers *without* current suicidal ideation; as well as treat and thereby *reduce* suicidal ideation severity among asylum seekers *with* current suicidal ideation. Finally, we tested whether the expected prevention and intervention effects of the mindfulness- and compassion-based intervention for suicidal ideation onset and severity were mediated by therapeutic effects of MBTR-R on stress- and trauma-related mental health outcomes. See Supplementary Material for more details on our rationale to focus the current study on suicidal ideation.

## Study 1 method

### Participants

Three-hundred-fifty-five East African asylum seekers from Eritrea and Sudan (*M* (s.d.)age = 35.15 (8.29) years) who sought refuge in Israel were recruited from the community between August 2013 to May 2019 in Tel Aviv, Israel. The sample included 116 Sudanese participants who completed assessments in Arabic and 239 Eritrean participants who completed assessments in Tigrinya. On average, participants had lived in Israel for 4 years (*M* (s.d.)_post-displacement time-in-Israel_ = 3.91 (3.39)). Currently, in Israel, less than 0.5% of the asylum requests are recognised (Orr and Ajzenstadt, 2020) and none of the participants had recognised refugee status. They have a ‘group protection’ status which functionally entitles them only the temporary right not to be deported.

### Procedure

Participants were recruited directly from the community of asylum seekers in Israel, via flyers, local non-governmental and municipal organisations. See Supplementary Material for more details on participants and community recruitment.

### Measures

Measures were translated and back-translated to Tigrinya and Arabic and psychometrically evaluated and validated for this study or in earlier research (Badri *et al*., 2012; Reebs *et al*., 2017; Yuval and Bernstein, 2017; Yuval *et al*., 2021). All measures were pilot-tested among Sudanese (Arabic) and Eritrean (Tigrinya) asylum seekers and revised, in an iterative process, which included cognitive interviewing with translators and asylum seekers to ensure linguistic and socio-cultural meaning (Sartorius and Kuyken, 1994; Miller and Fernando, 2008).

The *Harvard Trauma Questionnaire* (HTQ; Mollica *et al*., 1992) was used to measure traumatic stress exposure and PTSD symptoms. The *Brief Patient Health Questionnaire* (PHQ-9; Spitzer *et al*., 1999) was used to measure suicidal ideation and depression symptoms. See Discussion section for expanded discussion and rationale for this measurement approach to suicidal ideation in this population and post-displacement setting. The *Beck Anxiety Inventory* (BAI; Beck *et al*., 1988; Norman *et al*., 2006) was used to measure levels of anxiety symptoms. Using the categorical (diagnostic) symptom status for PTSD, depression and anxiety, we computed a *comorbidity index* (0 = no psychiatric symptomatology, 1 = uni-morbid or diagnostic symptom levels in one condition, 2 = co-morbid or diagnostic symptom levels in two conditions, 3 = multi-morbid or diagnostic symptom levels in all three conditions). Finally, the *Post-Migration Living Difficulties Scale* (Silove *et al*., 1997) was used to measure current post-migration stressors. See Supplementary Material for detailed information on measures and scoring.

### Results

First, point-prevalence of suicidal ideation was 31.0% (*n* = 108). Among the sub-sample endorsing current suicidal ideation (*n* = 108, *M* (s.d.) = 1.63 (0.82)), 58.3% (*n* = 63) reported suicidal thoughts several days/week, 20.4% (*n* = 22) suicidal thoughts more than half the days/week and 21.3% (*n* = 23) reported suicidal thoughts nearly every day. Men (28.8%, *M* (s.d.) = 0.45 (0.84)) and women (35.7%, *M* (s.d.) = 0.62 (0.96)) did not report different rates or severity of suicidal ideation (*t*(346) = −1.61, *p* = 0.11).

Second, in multiple regression analyses, post-migration stressor severity and trauma history exposure, together, explained a significant although relatively small proportion of variance in suicidal ideation severity. Whereas post-migration stressor severity accounted for unique variance in suicidal ideation severity, trauma history exposure was not uniquely associated with suicidal ideation beyond post-migration stressor severity. Findings did not differ between men and women. See Table 1.

**Table 1.** Linear regression of risk markers and factors predicting suicidal ideation severity (study 1 (*N* = 355))

	*F*	df	*p*	*R*^2^	*t*	*p*	*β*	*r*	sr*^2^*
Risk markers									
Total model	21.03	2342	0.00	0.11					
Post-migration stressor severity					5.68	0.00	0.30	0.33	0.08
Trauma history exposure					0.75	0.45	0.03	0.16	0.001
Risk factors									
PTSD	79.27	1345	0.00	0.19					
Anxiety	131.93	1296	0.00	0.31					
Depression	137.91	1382	0.00	0.29					
Multi-morbidity	115.89	2346	0.00	0.25					

**Fig. 1.**
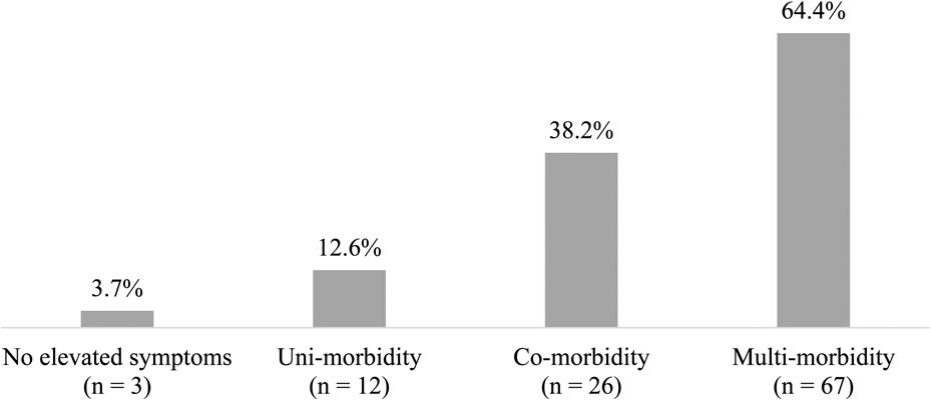
Rates of suicidal ideation by level of multi-morbidity. *Note:* Number and percentage of participants endorsing suicidal ideation as a function of level of multi-morbidity.

See Fig. 1 for rates of suicidal ideation as a function of degree of multi-morbidity. In logistic regression analyses, participants with *v*. without PTSD, with *v*. without depression, as well as with *v*. without elevated anxiety symptoms, were significantly more likely to report suicidal ideation. Participants exhibiting greater levels of multi-morbidity of PTSD, depression and anxiety (0 *v*. 1 *v*. 2 *v*. 3 elevated syndromes) were also significantly more likely to report suicidal ideation (*χ*^2^(2) = 107.05, *p* = 0.000, *B* (s.e.) = 1.26, OR = 3.54, 95% CI [2.66–4.72]). In linear regressions, we found that, PTSD, depression and anxiety each explained a large and significant proportion of variance in suicidal ideation severity. Likewise, degree of multi-morbidity explained a large and significant proportion of variance in suicidal ideation severity. These associations did not differ between men and women. See Table 1.

## Study 2 method

### Participants

This study was a secondary analysis of a single-site randomised control trial examining MBTR-R *v*. a waitlist control in a community sample of 158 (46% female) unrecognised and traumatised Eritrean asylum seekers residing in a high-risk, unstable urban post-migration setting in the Middle East (Israel). As noted, study 2 participants are a sub-sample of the study 1 sample. See Supplementary Material for more information on sampling and participants.

### Procedure

Following assessment for eligibility to participate in the study through a phone screening, consent and randomisation to condition (see Consort Diagram in Supplementary Material), participants completed the pre-intervention assessments. Following the 9-week intervention or identical waitlist-control period, participants completed a post-intervention assessment. Participants randomised to MBTR-R also completed a follow-up assessment 5 weeks after the post-intervention assessment. Waitlist-control participants only completed the 9-week post-waitlist assessment – to ensure that we did not unnecessarily withhold treatment for asylum seekers in the waitlist-control condition (Gold *et al*., 2017). See Supplementary Material for more details on the MBTR-R intervention and waitlist-control condition. See study 1 and Supplementary Material for details on measures.

### Results

#### Aim I: prospective stability of suicidal ideation

First, among participants in the waitlist-control with suicidal ideation at baseline (*n* = 26), 86.4% (*n* = 19) still endorsed suicidal ideation at post-waitlist assessment. Second, among participants in the waitlist control without suicidal ideation at baseline (*n* = 36), 23.1% (*n* = 6) endorsed suicidal ideation at post-waitlist assessment. Thus, 9-week prospective stability of suicidal ideation severity among participants in the waitlist-control from baseline (*M* (s.d.) = 0.66 (0.87), *n* = 48) to post-waitlist assessment (*M* (s.d.) = 0.81 (0.96), *n* = 48) was moderate (*κ* = 0.537, 95% CI [0.351–0.723], *p* < 0.000) (Altman, 1990).

#### Aim II: prospective prediction of the onset and severity of suicidal ideation

Second, we conducted multi-level models in R (‘lmerTest’; Kuznetsova *et al*., 2017) to test prospective prediction of the onset and severity of suicidal ideation at 9-week post-waitlist assessment. Among participants in the waitlist-control without suicidal ideation at baseline (*n* = 35), post-migration stressors and trauma history exposure, together, did not prospectively predict suicidal ideation onset and severity at post-waitlist assessment (*β* = 0.54, s.e. = 0.53, *t* = 1.03, *p* = 0.31). However, PTSD (*β* = 0.64, s.e. = 0.16, *t* = 4.03, *p* < 0.001), depression (*β* = 0.08, s.e. = 0.02, *t* = 4.72, *p* < 0.001) and anxiety (*β* = 0.43, s.e. = 0.12, *t* = 3.55, *p* < 0.001), each prospectively predicted suicidal ideation onset and severity at post-waitlist assessment. Likewise, degree of multi-morbidity at baseline prospectively predicted suicidal ideation onset and severity at post-waitlist assessment (*β* = 0.39, s.e. = 0.10, *t* = 3.96, *p* < 0.001).

#### Aim III: prevention effects of MBTR-R on suicidal ideation

Third, we conducted multi-level models in R to prospectively predict the onset and level of suicidal ideation severity at post-intervention assessment and 5-week follow-up. Among all participants without suicidal ideation at baseline (*n* = 109), MBTR-R, relative to waitlist-control, prevented the onset and severity of suicidal ideation at post-intervention (model *R*^2^ = 0.19, *β* = −0.39, s.e. = 0.18, *t* = −2.10, *p* = 0.03) and follow-up (model *R*^2^ = 0.23, *β* = −0.21, s.e. = 0.09, *t* = −2.37, *p* = 0.02). Specifically, whereas 23.1% of waitlist-controls endorsed suicidal ideation at post-waitlist assessment, 15.6% of MBTR-R participants endorsed suicidal ideation at post-intervention assessment and 9.8% at follow-up. The observed preventive effect of MBTR-R on suicidality onset and severity did not differ between men and women.

#### Aim IV: intervention effects of MBTR-R on suicidal ideation

Fourth, we conducted multi-level models in R to test the intervention effects of MBTR-R at post-intervention assessment and 5-week follow-up. Among all participants endorsing suicidal ideation at baseline (*n* = 46), MBTR-R, relative to waitlist-control, was not associated with lower levels of suicidal ideation severity at post-intervention assessment (model *R*^2^ = 0.18, *β* = −0.32, s.e. = 0.25, *t* = −1.26, *p* = 0.21) or follow-up (model *R*^2^ = 0.20, *β* = −0.23, s.e. = 0.14, *t* = −1.63, *p* = 0.11). Specifically, whereas 86.4% of waitlist-controls still endorsed suicidal ideation at post-waitlist assessment, 82.4% of MBTR-R participants still endorsed suicidal ideation at post-intervention assessment and 80% at follow-up. Notably, there was no treatment effect of MBTR-R on suicidal ideation severity among either men or women.

#### Aim V: does trauma recovery mediate prevention effect of MBTR-R on suicidal ideation?

We used a multi-level accelerated boot-strapped cross-product test of mediation in R (‘mediation’; Tingley *et al*., 2014) to test whether change from pre-to-post intervention in PTSD, depression and anxiety symptom severity mediated the effect of MBTR-R, relative to wait-list, on suicidal ideation onset and severity. We used restricted maximum likelihood to account for missing observations. Analyses were conducted among the Full Case Complete Intent-To-Treat (ITT) sample (see CONSORT in SM). See Table 2 for mediation model pathways. Among participants without suicidal ideation at baseline (*n* = 109), change in PTSD, depression, anxiety and level of multi-morbidity – from baseline to post-intervention assessment – each significantly mediated the observed preventive effect of MBTR-R, relative to waitlist-control, on suicidal ideation onset and severity. Because of the null intervention effect of MBTR-R relative to wait-list control (Aim IV), a test of mediation was not conducted among participants endorsing suicidality at baseline.

**Table 2.** Indirect effect of trauma- and stress-related mental health outcomes on the preventive effects of MBTR-R on suicidal ideation (study 2 (*N* = 109))

	ACME	95% CI	*P*	Proportion mediated	95% CI	*p*
PTSD	−0.13	[−0.23 to −0.04]	0.004	0.56	[0.18–1.78]	0.015
Depression	−0.13	[−0.24 to −0.02]	0.017	0.54	[0.13–1.44]	0.024
Anxiety	−0.13	[−0.24 to −0.03]	0.009	0.56	[0.18–1.65]	0.014
Multi-morbidity	−0.12	[−0.23 to −0.02]	0.015	0.53	[0.12–1.47]	0.028

*Note:* ACME, average causal mediation effect.

## Discussion

There are growing concerns about a global public health crisis of suicidality among refugees and asylum seekers (Vijayakumar, 2016; Haroz *et al*., 2020). Study of suicidality and suicidal ideation specifically, as well as its prevention and intervention, particularly in fast-growing high-risk unstable post-displacement settings wherein the large majority of FDPs currently reside is scarce, but much needed (Aichberger, 2014; Colucci *et al*., 2017; Ventevogel *et al*., 2019). We, therefore, sought to estimate prevalence, associated risk factors and prospective stability of suicidal ideation. Moreover, we sought to test the capacity of a mindfulness- and compassion-based intervention program (MBTR-R; Aizik-Reebs *et al*., 2021) to prevent the onset, as well as reduce the severity of pre-existing suicidal ideation in a community sample of unrecognised East African asylum seekers in a high-risk unstable post-migration setting in the Middle East (Israel).

First, observed point-prevalence and severity of suicidal ideation in this general community sample of asylum seekers were high (31%) and comparable to previously reported estimates in refugee camps and even select clinical samples of refugees seeking mental health treatment (Rahman and Hafeez, 2003; Ssenyonga *et al*., 2013; Lerner *et al*., 2016; Premand *et al*., 2018). Observed levels of suicidal ideation are consistent with, and likely a function of, elevated post-migration stressors in this urban, unstable post-displacement context and related stress- and trauma-related mental health problems (Li *et al*., 2016; Giacco *et al*., 2018). Indeed, we observed a significant cross-sectional association between post-migration living difficulties, post-traumatic stress, depression, anxiety symptoms and their multi-morbidity with suicidal ideation. Notably, trauma exposure severity was not uniquely associated with suicidal ideation beyond post-migration stressor severity (Li *et al*., 2016; Priebe *et al*., 2016). Furthermore, depression symptom severity and multi-morbidity prospectively predicted the onset and severity of suicidal ideation, consistent with similar findings linking psychopathology severity and suicidal ideation among WEIRD populations (Mann *et al*., 2005).

Second, mindfulness- and compassion-based training tailored to diverse FDPs (Aizik-Reebs *et al*., 2021) successfully prevented the onset of suicidal ideation. This preventive effect was mediated by therapeutic effects of the intervention on stress- and trauma-related mental health outcomes. In contrast, intervention effects of MBTR-R to reduce current levels of suicidal ideation were not observed. Thus, MBTR-R therapeutically impacted suicidal ideation by preventing its onset but not by facilitating its remission. These initial findings are consistent with earlier empirical findings documenting the preventive effects of MBCT on depression relapse and suicidal cognition in WEIRD populations (Forkmann *et al*., 2014; Barnhofer *et al*., 2015). These are novel, albeit preliminary, findings with respect to preventive effects of MBIs on suicidal ideation in the context of trauma recovery broadly and among FDPs specifically. Likewise, findings are consistent with theory and previous findings that stress- and trauma-related mental health outcomes function as malleable causal risk factors for suicidality, and as likely important therapeutic targets for suicide prevention (Mann *et al*., 2005; Vijayakumar, 2016). These initial, albeit promising, preventive effects of MBTR-R are noteworthy given limited study or interventions to prevent suicidal ideation among FDPs (Barbui *et al*., 2020; Tol *et al*., 2020). Moreover, MBIs like MBTR-R may be particularly suitable for implementation and scaling-up in high-risk post-displacement settings. They are brief, group-based, low-cost and beneficial to participants with a range of stress-related distress and personal goals (Singla *et al*., 2017); and have been adapted to a variety of populations and contexts in ways that are socio-culturally sensitive to diverse backgrounds, belief systems and languages (Hinton *et al*., 2013; Crane *et al*., 2017).

The study is also limited in a number of ways. First, the study was conducted among one community sample of East African asylum seekers residing in Israel. This sampling strategy permits robust socio-cultural adaptation of MBTR-R to this population per best-practices in global mental health interventions (Kirmayer *et al*., 2017; Singla *et al*., 2017), and may buffer against potential threats of internal validity emerging from ad-mixing of different refugee populations (Kirmayer *et al*., 2017; Yuval and Bernstein, 2017; Yuval *et al*., 2021). Yet, it is important that future work examine whether observed findings generalise to other refugee populations and contexts. Second, asylum seekers in study 2 were screened and 21 participants were excluded based on active suicidality. Thus, observed rates of suicidal ideation likely slightly *underestimate* the actual prevalence of suicidal ideation in this population. Moreover, by design based on pilot testing of alternative measurement methods, measurement of suicidal ideation was limited to one item of the PHQ-9 depression questionnaire. Our pilot work and previous research in this context indicated that due to social and religious stigma of suicide in this asylum-seeker population, repeated (e.g. multiple questions related to cognitions or behaviour) and more interpersonally direct inquiry (e.g. structured interviews) is likely to bias and systematically underestimate rates of suicidal ideation (Kashyap and Joscelyne, 2020). Yet, we also, *a priori*, recognise that although this measure of suicidal ideation may be sensitive to predict future suicidal behaviour, it is not specific and may over-estimate risk of suicide for some asylum seekers (Razykov *et al*., 2012; Na *et al*., 2018). From public health and clinical ethics perspectives, we believe that it is far more costly to miss caseness or severity of suicidal ideation than it is to detect suicidal ideation that may not lead to suicidal behaviour (Vijayakumar, 2016; Jobes and Joiner, 2019). We also speculate that it may be important for future studies among diverse FDPs to more systematically study epidemiologic estimates of suicidal ideation and suicidal behaviour as a function of multi-method multi-modal measurement (Hopwood and Bornstein, 2014). Third, because of uncertainty regarding residential status of this population of asylum seekers at the time of the study (Guthmann, 2018) and the logistical complexity of following asylum seeker participants in unstable high-risk post-displacement context over time (Carlsson *et al*., 2014; Troup *et al*., 2021), the study tested prospective stability of suicidal ideation and intervention effects of MBTR-R over a relatively short 9-week period. Due to censored prospective observations, detecting preventive effects was systematically less likely as a result, particularly in this modest sample size. Future research should test the stability of suicidal ideation as well as maintenance of observed prevention effects of MBTR-R over a longer time period (Priebe *et al*., 2016). Finally, observed effects need to be replicated, tested in larger samples and relative to more rigorous randomised active-controlled interventions (Carlsson *et al.*, 2014).

The present findings may have a number of implications. Findings point to the potential significance and urgency of a global public health crisis of suicidality among FDPs, particularly in high-risk unstable urban post-displacement settings wherein the majority of refugees and asylum seekers currently reside worldwide. In addition, findings illustrate the potential importance of investment in research dedicated to early detection, prevention and intervention targeting suicidal ideation among high-risk FDPs (Aichberger, 2014; Ventevogel *et al*., 2019). Likewise, findings may inform post-displacement municipal, state and national policy-making to impact mental health and related suicidality outcomes (WHO, 2014); Priebe *et al.*, 2016). Indeed, findings indicate that mindfulness- and compassion-based training tailored to diverse FDPs may help to prevent suicidal ideation through facilitating trauma recovery.

## Data Availability

The data that support the findings of this study are available from the corresponding author upon reasonable request.

## Supplementary material

For supplementary material accompanying this paper visit https://doi.org/10.1017/S2045796022000579. click here to view supplementary material
