# Supplementary material for: Prevalence and prevention of suicidal ideation among asylum seekers in a high-risk urban post-displacement setting
Source: Epidemiol Psychiatr Sci. 2022 Oct 17;31:e76. doi: 10.1017/S2045796022000579 (PMC9583629; doi:10.1017/S2045796022000579)
Supplement: Supplementary file 1 [file S2045796022000579sup001.doc]

# Supplemental Materials for “Prevalence and Prevention of Suicidal Ideation among Asylum Seekers in a High-Risk Urban Post-Displacement Setting”

Aizik-Reebs, A., Yuval, K., Beyene Kesete, Y., Lurie, I., & Bernstein, A.

**Introduction Supplement**

In these studies, we focused on suicidal ideation for three key reasons. First, suicidal ideation is likely to be the most reliable epidemiological estimate of suicidality in these populations and post-displacement contexts (WHO 2014; Kashyap and Joscelyne 2020). The quality of epidemiological estimates of suicidal behaviors in these contexts is likely questionable for a number of reasons (WHO 2014; Bachmann 2018), including illegality of suicidal behaviors in most of these post-displacement contexts, misclassification, religious conventions, and common socio-cultural stigmata associated with suicidal behavior (Rahman and Hafeez 2003; Gearing and Alonzo 2018). Second, suicidal ideation lawfully precedes suicidal behavior, is systematically related to risk for suicidal behavior, is malleable, and thus is a powerful intervention target to prevent suicidal behavior (O'Connor and Nock 2014; Jobes and Joiner 2019; Van Orden et al. 2010; Silverman et al. 2007). Third, focus on suicidal ideation facilitates more statistically-powered prevention and intervention studies. In contrast, reliance on attempts or death by suicide – much lower base-rate phenomena – requires much larger and less feasible studies particularly in complex and unstable post-displacement contexts (Jobes and Joiner 2019; Silove, Ventevogel, and Rees 2017; Christensen, Cuijpers, and Reynolds 2016). Fourth, suicidal ideation may be a relatively non-specific predictor of suicidal behavior, yet must be considered relative to our limited capacity to predict suicidality to begin with. For example, a large recent meta-analysis found that candidate predictors of suicide are, in fact, relatively weak, and among them, suicidal ideation is the third most powerful predictor of death by suicide (Franklin et al. 2017). It is also critical to highlight that, clinically, suicidal ideation is a highly concerning phenomenon in and of itself – a salient marker of distress and desperation linked to stress and trauma (Jobes and Joiner 2019; Kroenke, Spitzer, and Williams 2001). Thus, despite these inherent limitations, suicidal ideation may be a particularly important epidemiological indicator of potential suicidality as well as intervention target and outcome among refugees and asylum seekers in high-risk post-displacement settings (Vijayakumar 2016; Colucci, Too, and Minas 2017).

**Method Supplement**

**Participants**

The selected sample of Eritrean asylum seekers are representative of a large and fast-growing population of forcibly displaced people in the current global refugee crisis (UNHCR 2019). First, members of this community were exposed to a large number of potentially traumatic events including serious violations of human rights, arbitrary detention, torture, sexual and gender-based violence, religious and political persecution (Connell 2012; Van Reisen and Mawere 2017). They fled from a highly repressive state and compulsory military service in Eritrea, violations of human rights, arbitrary detention, enforced disappearances, sexual and gender-based violence, religious and political persecution; and then while fleeing from their home country, a large percent of this community were survivors of human trafficking and torture in the Egyptian Sinai desert (Nakash et al. 2015; United Nations High Commissioner for Refugees 2016; Connell 2012). Furthermore, this population of African refugees residing in Israel is representative of the millions of African refugees who have been forcibly displaced throughout Europe in recent years (UNHCR 2015; United Nations 2015). East African refugees constitute the largest refugee population from and in Africa and are one of the largest refugee populations world-wide; Sudan (including South Sudan) and Eritrea are among the top 10 major source countries of refugees world-wide (UNHCR 2014, 2016, 2015). Second, members of this community have not received refugee or formal residential status or protections such that their future remains unpredictable and uncertain due to threat of detention or deportation (Rozen 2015; Orgal, Liberman, and Avivi 2019). Third, members of this community are struggling with chronic and often severe post-migratory life-stressors implicated in stress-related mental health problems that interfere with trauma recovery yet only a tiny fraction receive any mental health care let alone evidence-based care (Miller and Rasmussen 2017; Giacco, Laxhman, and Priebe 2018; Li, Liddell, and Nickerson 2016; Yuval et al. 2021). Their ongoing chronic migrant status instability, future uncertainty, and post-migratory life-stressors represent a fast-growing population of forcibly displaced people worldwide (UNHCR 2019; Patel et al. 2018).

**Community Recruitment**

Both studies received human subjects research ethics approval by a University of Haifa Institutional Review Board committee. Participants were recruited via public flyers, community recruitment and via local NGOs and municipal organizations working with refugees. As part of standard clinical practice and to mitigate mistrust that asylum seekers harbor towards local state institutions, candidate participants were ensured that confidentiality and anonymity would not be breached. Participants completed informed verbal and written consent in their native language. Participants received ~15$ USD per hour in exchange for their participation. To help ensure broad recruitment and participation, data were collected in an ambulatory laboratory office space in the heart of the asylum seeker community in South Tel Aviv.

**Sampling: Study 2**

Over the course of one year, male and female Eritrean asylum seekers were recruited in three cohorts, and randomized to either MBTR-R or waitlist control. Exclusion criteria were (a) past suicide attempt or at acute risk for committing suicide (21 out of 200 screened participants were excluded due to suicidal behaviors, see Consort Diagram below for more details), (b) current psychotic symptoms, (c) current mental health treatment: psychiatrist, psychotherapy, psycho-social support group. Randomization was conducted via random number generation in blocks of two conditions with a ratio of three MBTR-R participants to two waitlist control participants. This was done based on a power analysis to, first, ensure sufficient number of participants to detect medium size between-group effects; and, second, to ensure sufficient power to detect moderate effects in planned within-group analyses among the MBTR-R group (Borm et al, 2007; Moher et al, 2009).

**Measurements**

The *Harvard* *Trauma Questionnaire* (HTQ; (Mollica et al. 1992) was used to measure traumatic stress exposure as well as PTSD symptoms. HTQ was developed to be used and adapted across socio-cultural groups and languages, and thus is a well-established instrument to measure traumatic stress and PTSD symptoms in diverse forcibly displaced populations, including E. African populations specifically (Darzi 2017; Nakeyar and Frewen 2016; Hollifield et al. 2002; Reebs, Yuval, and Bernstein 2017). HTQ mean cut-off score ≥ 2 is commonly used to identify categorical (diagnostic) symptom status of PTSD (Silove et al. 2007; Oruc et al. 2008; Tinghög et al. 2017). As in past studies, HTQ trauma history exposure was computed based on the mean levels of exposure (i.e., 1 = not exposed, 2 = heard about, 3 = witnessed, 4 = experienced) to each of the 16 traumatic events relevant to refugees’ experiences (Yuval, Zvielli, and Bernstein 2016).

The *Brief Patient Health Questionnaire* (PHQ-9; (Spitzer, Kroenke, and Williams 1999) was used to measure suicidal ideation as well as depression symptoms. In particular, item 9, “thoughts that you would be better off dead, or of hurting yourself,” was used to assess suicidal ideation over the past week. To identify point-prevalence of suicidal ideation, participants’ response to item 9 was classified as endorsing suicidal ideation (“several days” or “more than half the days” or “nearly every day”) and denying ideation if responding (“not at all”). Item 9 of the PHQ-9 has widely been used as a brief highly sensitive measure of suicidal ideation (Walker et al. 2011; Walker et al. 2008; Bauer et al. 2013). Across multiple large studies and samples, endorsing PHQ-9 suicidal ideation has correlated with elevated risk of suicidal behavior in the general population, clinical samples and among people exposed to traumatic events (Louzon et al. 2016; Simon et al. 2013; Rossom et al. 2017). Yet, the predictive value of this single-item index of suicidal ideation for suicide risk has been questioned in studies comparing it to diagnostic interviews (Na et al. 2018; Razykov et al. 2012). See Discussion section for expanded discussion and rationale for this specific measurement approach to suicidal ideation in this FDP population and post-displacement setting. To identify categorical (diagnostic) symptom status of depression, a PHQ cut-off score ≥10 is commonly used (Manea, Gilbody, and McMillan 2012). The PHQ-9 is a commonly used measure of depression in diverse populations and refugee populations (Poole et al. 2019).

The *Beck Anxiety Inventory* (BAI; (Beck et al. 1988)) was used to measure levels of anxiety symptoms. BAI has been commonly used as a self-report tool to measure anxiety, also among refugee populations (Alexander, David, and Grills 2013; Turner et al. 2003). BAI total cut-off score ≥ 16 is commonly used to identify categorical (diagnostic) symptom status of anxiety disorder (Beck and Steer 1993; Bardhoshi, Duncan, and Erford 2016).

Using the categorical (diagnostic) symptom status for PTSD, depression, and anxiety, we computed a *comorbidity index* (0 = no psychiatric symptomatology, 1 = uni-morbid or diagnostic symptom levels in one condition, 2 = co-morbid or diagnostic symptom levels in two conditions, 3 = multi-morbid or diagnostic symptom levels in all three conditions).

Finally, the *Post-Migration Living Difficulties Scale* (Silove et al. 1997) was used to measure current post-migration stressors. The *PMLDS* has been widely applied to measure post-migration stressors across a variety of refugee and migrant populations (Li, Liddell, and Nickerson 2016; Schick et al. 2018).

**Procedure**

***MBTR-R Intervention Condition.*** MBTR-R is a mindfulness-based group (10-20 participants) intervention consisting of nine 2.5-hour weekly sessions. MBTR-R format and structure parallel common MBIs (Crane et al. 2017) including Mindfulness Based Stress Reduction (MBSR) and Mindfulness Based Cognitive Therapy (MBCT) (Kabat-Zinn 2017; Segal, Williams, and Teasdale 2013). Key trauma-sensitive adaptations to mindfulness meditation practices were included in MBTR-R to prevent difficulties during mindfulness meditation as well as to reduce risk of adverse responding and to optimize salutary benefits (Treleaven 2018). To provide optimal conditions for participants to learn mindfulness and key intervention principles and to benefit from the group format, delivery of MBTR-R was socio-culturally adapted (see (Aizik-Reebs et al. 2021) for more details).

***Waitlist-control Condition.*** Following the 9-week waitlist period and post-waitlist assessment, participants randomized to waitlist-control were offered an equivalent group intervention (i.e., 22.5 total hours, group instructor and cultural mediator, psychoeducation and low-intensity cognitive behavior therapy skill training, relaxation techniques).

**Figure 1**

**Assessed for eligibility** **in phone interview (N = 200)**

*Consort Diagram*

**Excluded (n = 42)**

**Not meeting inclusion criteria**

♦ Psychosis (n = 14)

♦ Acute suicidality (n = 3)

♦ Past suicide attempt (n = 13)

♦ Past suicidality & psychosis (n = 5)

♦ Receiving mental health treatment (n = 5)

♦ Participating in support group (n = 2)

**Randomized (N = 158)**

**Randomized to Control Group &**

**Completed Pre-Assessment (n = 60)**

**Randomized to MBTR-R Group & Completed Pre-Assessment (n = 98)**

**Did not receive allocated intervention (n = 20)**

♦ Moved away/detained (n = 4)

♦ No time to participate (n = 1)

♦ No longer interested to participate (n = 15)

**Received at least one session**

**of allocated intervention** **(n = 78)**

**Did not complete Post-assessment (n = 12)**

♦ No time for assessment (n= 6)

♦ Moved away/detained (n = 2)

♦ Lost contact (n = 4)

**Did not complete Post- assessment**

**(n = 9)**

♦ No time for assessment (n = 2)

♦ Moved away/detained (n = 1)

♦ Lost contact (n = 6)

**Did not complete Post-assessment (n = 6)**

♦ No time for assessment (n = 1)

♦ Moved away/detained (n = 3)

♦ Lost contact (n = 2)

**Completed Post-assessment** **(n = 72)**

**Completed Post-assessment** **(n = 48)**

**Completed Post-assessment** **(n = 11)**

**Did not complete Follow-Up Assessment (n = 16)**

♦ Lost contact (n = 2)

♦ Assessed only at post-assessment due to limited intervention dose (attended < 2 MBTR-R sessions) (n = 14)

**Completed Follow-Up (n = 56)**

**Analysed** 

♦ Intervention completers analysis (n = 52)

♦ Full Case Complete Intent-to-Treat analysis (n = 83)

**Analysed** 

♦ Intervention completers analysis (n = 48)

♦ Full Case Complete Intent-to-Treat analysis

(n = 48)

**References**

Aizik-Reebs, Anna, Kim Yuval, Yuval Hadash, Solomon Gebreyohans Gebremariam, and Amit Bernstein. 2021. 'Mindfulness-Based Trauma Recovery for Refugees (MBTR-R): Randomized Waitlist-Control Evidence of Efficacy and Safety', *Clinical Psychological Science*, 9: 1164-84.

Alexander, Binu, Elizabeth David, and Nathan Grills. 2013. 'High prevalence of anxiety disorders among adolescent Tibetan refugees', *Asian Journal of Psychiatry*, 6: 218-21.

Bachmann, Silke. 2018. 'Epidemiology of Suicide and the Psychiatric Perspective', *International Journal of Environmental Research and Public Health*, 15.

Bardhoshi, Gerta, Kelly Duncan, and Bradley T. Erford. 2016. 'Psychometric Meta-Analysis of the English Version of the Beck Anxiety Inventory', *Journal of Counseling & Development*, 94: 356-73.

Bauer, Amy M., Ya-Fen Chan, Hsiang Huang, Steven Vannoy, and Jürgen Unützer. 2013. 'Characteristics, management, and depression outcomes of primary care patients who endorse thoughts of death or suicide on the PHQ-9', *Journal of General Internal Medicine*, 28: 363-69.

Beck, Aaron T., Norman Epstein, Gary Brown, and Robert A. Steer. 1988. 'An inventory for measuring clinical anxiety: Psychometric properties', *Journal of Consulting and Clinical Psychology*, 56: 893-97.

Beck, Aaron T., and Robert A Steer. 1993. *BAI: Beck anxiety inventory manual* (Psychological Corporation: San Antonio).

Christensen, Helen, Pim Cuijpers, and Charles F. Reynolds, III. 2016. 'Changing the Direction of Suicide Prevention Research: A Necessity for True Population Impact', *JAMA psychiatry*, 73: 435-36.

Colucci, Erminia, Lay San Too, and Harry Minas. 2017. 'A suicide research agenda for people from immigrant and refugee backgrounds', *Death Studies*, 41: 502-11.

Connell, Dan. 2012. 'Escaping Eritrea: Why They Flee and What They Face', *Middle East Report*: 2-9.

Crane, Rebecca S., J. Brewer, C. Feldman, J. Kabat-Zinn, S. Santorelli, J. M. G. Williams, and W. Kuyken. 2017. 'What defines mindfulness-based programs? The warp and the weft', *Psychological Medicine*, 47: 990-99.

Darzi, Chantal. 2017. 'The Harvard Trauma Questionnaire: Reliability and Validity Generalization Studies of the Symptom Scales', University of Ottawa.

Franklin, Joseph C., Jessica D. Ribeiro, Kathryn R. Fox, Kate H. Bentley, Evan M. Kleiman, Xieyining Huang, Katherine M. Musacchio, Adam C. Jaroszewski, Bernard P. Chang, and Matthew K. Nock. 2017. 'Risk factors for suicidal thoughts and behaviors: A meta-analysis of 50 years of research', *Psychological Bulletin*, 143: 187-232.

Gearing, Robin Edward, and Dana Alonzo. 2018. 'Religion and Suicide: New Findings', *Journal of Religion and Health*, 57: 2478-99.

Giacco, Domenico, Neelam Laxhman, and Stefan Priebe. 2018. 'Prevalence of and risk factors for mental disorders in refugees', *Seminars in Cell & Developmental Biology*, 77: 144-52.

Hollifield, Michael, Teddy D. Warner, Nityamo Lian, Barry Krakow, Janis H. Jenkins, James Kesler, Jayne Stevenson, and Joseph Westermeyer. 2002. 'Measuring trauma and health status in refugees: A critical review', *JAMA: Journal of the American Medical Association*, 288: 611-21.

Jobes, David A., and Thomas E. Joiner. 2019. 'Reflections on Suicidal Ideation', *Crisis*, 40: 227-30.

Kabat-Zinn, Jon. 2017. *Mindfulness-Based Stress Reduction (MBSR). Authorized Curriculum Guide* (Center for Mindfulness in Medicine, Health Care, and Society (CFM), University of Massachusetts Medical School ).

Kashyap, Shraddha, and Amy Joscelyne. 2020. 'Refugees and suicide: when the quest for a better life becomes thwarted.' in Andrew Page and Werner Stritzke (eds.), *Alternatives to Suicide* (Elsevier).

Kroenke, Kurt, Robert L. Spitzer, and Janet B. W. Williams. 2001. 'The PHQ-9: Validity of a Brief Depression Severity Measure', *Journal of General Internal Medicine*, 16: 606-13.

Li, Susan SY, Belinda J Liddell, and Angela Nickerson. 2016. 'The Relationship Between Post-Migration Stress and Psychological Disorders in Refugees and Asylum Seekers', *Current psychiatry reports*, 18: 82.

Louzon, Samantha A. , Robert Bossarte, John F. McCarthy, and Ira R. Katz. 2016. 'Does Suicidal Ideation as Measured by the PHQ-9 Predict Suicide Among VA Patients?', *Psychiatric Services*, 67: 517-22.

Manea, Laura, Simon Gilbody, and Dean McMillan. 2012. 'Optimal cut-off score for diagnosing depression with the Patient Health Questionnaire (PHQ-9): a meta-analysis', *CMAJ : Canadian Medical Association journal = journal de l'Association medicale canadienne*, 184: E191-E96.

Miller, K. E., and A. Rasmussen. 2017. 'The mental health of civilians displaced by armed conflict: an ecological model of refugee distress', *Epidemiology and Psychiatric Sciences*, 26: 129-38.

Mollica, Richard F, Yael Caspi-Yavin, Paola Bollini, Toan Truong, Svang Tor, and James Lavelle. 1992. 'The Harvard Trauma Questionnaire: validating a cross-cultural instrument for measuring torture, trauma, and posttraumatic stress disorder in Indochinese refugees', *The Journal of nervous and mental disease*, 180: 111-16.

Na, Peter J., Satyanarayana R. Yaramala, Jihoon A. Kim, Hyelee Kim, Fernando S. Goes, Peter P. Zandi, Jennifer L. Vande Voort, Bruce Sutor, Paul Croarkin, and William V. Bobo. 2018. 'The PHQ-9 Item 9 based screening for suicide risk: a validation study of the Patient Health Questionnaire (PHQ)−9 Item 9 with the Columbia Suicide Severity Rating Scale (C-SSRS)', *Journal of Affective Disorders*, 232: 34-40.

Nakash, Ora, Maayan Nagar, Anat Shoshani, and Ido Lurie. 2015. 'The association between acculturation patterns and mental health symptoms among Eritrean and Sudanese asylum seekers in Israel', *Cultural Diversity and Ethnic Minority Psychology*, 21: 468.

Nakeyar, Cisse, and Paul A. Frewen. 2016. 'Evidence-based care for Iraqi, Kurdish, and Syrian asylum seekers and refugees of the Syrian civil war: A systematic review', *Canadian Psychology/Psychologie canadienne*, 57: 233-45.

O'Connor, Rory C., and Matthew K. Nock. 2014. 'The psychology of suicidal behaviour', *The Lancet Psychiatry*, 1: 73-85.

Orgal, Yael Agur, Gilad Liberman, and Sigal Kook Avivi. 2019. 'Israel’s ‘Voluntary’Return Policy to Expel Refugees: The Illusion of Choice', *Mobile Africa: Human Trafficking and the Digital Divide*: 209-37.

Oruc, Lilijana, Aida Kapetanovic, Naris Pojskic, Kate Miley, Sharon Forstbauer, Richard F. Mollica, and David C. Henderson. 2008. 'Screening for PTSD and depression in Bosnia and Herzegovina: validating the Harvard Trauma Questionnaire and the Hopkins Symptom Checklist', *International Journal of Culture and Mental Health*, 1: 105-16.

Patel, Vikram, Shekhar Saxena, Crick Lund, Graham Thornicroft, Florence Baingana, Paul Bolton, Dan Chisholm, Pamela Y Collins, Janice L Cooper, and Julian Eaton. 2018. 'The Lancet Commission on global mental health and sustainable development', *The Lancet*, 392: 1553-98.

Poole, Danielle, Shirley Liao, Elysia Larson, Bethany Hedt-Gauthier, Nathaniel Raymond, and Till Börnighausen. 2019. 'Implementation of a sequential screening process for depression in humanitarian crises: a validation study of the Patient Health Questionnaire in Syrian refugees', *The Lancet Global Health*, 7: S42.

Rahman, A., and A. Hafeez. 2003. 'Suicidal feelings run high among mothers in refugee camps: a cross-sectional survey', *Acta Psychiatrica Scandinavica*, 108: 392-93.

Razykov, Ilya, Roy C. Ziegelstein, Mary A. Whooley, and Brett D. Thombs. 2012. 'The PHQ-9 versus the PHQ-8 — Is item 9 useful for assessing suicide risk in coronary artery disease patients? Data from the Heart and Soul Study', *Journal of psychosomatic research*, 73: 163-68.

Reebs, Anna, Kim Yuval, and Amit Bernstein. 2017. 'Remembering and Responding to Traumatic Autobiographical Memories: Exploring Risk and Intervention Targets for Posttraumatic Stress in Traumatized Refugees', *Clinical Psychological Science*, 5: 789-97.

Rossom, Rebecca C., Karen J. Coleman, Brian K. Ahmedani, Arne Beck, Eric Johnson, Malia Oliver, and Greg E. Simon. 2017. 'Suicidal ideation reported on the PHQ9 and risk of suicidal behavior across age groups', *Journal of Affective Disorders*, 215: 77-84.

Rozen, Sigal. 2015. "Deported to the Unknown." In. Tel Aviv, Israel: Hotline for Refugees and Migrations.

Schick, Matthis, Naser Morina, Panagiota Mistridis, Ulrich Schnyder, Richard A. Bryant, and Angela Nickerson. 2018. 'Changes in Post-migration Living Difficulties Predict Treatment Outcome in Traumatized Refugees', *Frontiers in Psychiatry*, 9.

Segal, Zindel V., J. M. Williams, and John D. Teasdale. 2013. *Mindfulness-based cognitive therapy for depression (2nd ed.)* (Guilford Press: New York, NY).

Silove, Derrick, Vijaya Manicavasagar, Richard Mollica, Meng Thai, Dorani Khiek, James Lavelle, and Svang Tor. 2007. 'Screening for Depression and PTSD in a Cambodian Population Unaffected by War: Comparing the Hopkins Symptom Checklist and Harvard Trauma Questionnaire With the Structured Clinical Interview', *The Journal of nervous and mental disease*, 195: 152-57.

Silove, Derrick, Ingrid Sinnerbrink, Annette Field, Vijaya Manicavasagar, and Zachary Steel. 1997. 'Anxiety, depression and PTSD in asylum-seekers: assocations with pre-migration trauma and post-migration stressors', *The British Journal of Psychiatry*, 170: 351-57.

Silove, Derrick, Peter Ventevogel, and Susan Rees. 2017. 'The contemporary refugee crisis: an overview of mental health challenges', *World Psychiatry*, 16: 130-39.

Silverman, Morton M., Alan L. Berman, Nels D. Sanddal, Patrick W. O'Carroll, and Thomas E. Joiner. 2007. 'Rebuilding the Tower of Babel: A Revised Nomenclature for the Study of Suicide and Suicidal Behaviors. Part 1: Background, Rationale, and Methodology', *Suicide and Life-Threatening Behavior*, 37: 248-63.

Simon, Gregory E., Carolyn M. Rutter, Do Peterson, Malia Oliver, Ursula Whiteside, Belinda Operskalski, and Evette J. Ludman. 2013. 'Does Response on the PHQ-9 Depression Questionnaire Predict Subsequent Suicide Attempt or Suicide Death?', *Psychiatric Services*, 64: 1195-202.

Spitzer, R. L., K. Kroenke, and J. W. Williams. 1999. 'Validation and utility of a self-report version of prime-md: The phq primary care study', *JAMA*, 282: 1737-44.

Tinghög, Petter, Andreas Malm, Charlotta Arwidson, Erika Sigvardsdotter, Andreas Lundin, and Fredrik Saboonchi. 2017. 'Prevalence of mental ill health, traumas and postmigration stress among refugees from Syria resettled in Sweden after 2011: a population-based survey', *BMJ Open*, 7: e018899.

Treleaven, David A. 2018. *Trauma-sensitive mindfulness: Practices for safe and transformative healing* (W.W Norton & Company: New York).

Turner, W., C. Bowie, G. Dunn, L. Shapo, and W. Yule. 2003. 'Mental health of Kosovan Albanian refugees in the UK', *The British Journal of Psychiatry*, 182: 444-48.

UNHCR. 2014. "Sharp increase in number of Eritrean refugees and asylum-seekers in Europe, Ethiopia and Sudan." In.

———. 2015. "Asylum Levels and Trends in Industrialized Countries, 2014." In.

———. 2016. "Global Trends: Forced Displacement in 2015." In. Geneva, Switzerland: United Nations High Commissioner for Refugees.

———. 2019. "Global Trends: forced displacement in 2018." In. Geneva, Switzerland: United Nations High Commissioner for Refugees.

United Nations, Department of Economic and Social Affairs. 2015. "Trends in International Migrant Stock: Migrants by Destination and Origin " In.

United Nations High Commissioner for Refugees, UNHCR. 2016. "Global Trends: Forced Displacement in 2015." In. Geneva, Switzerland: UNHCR.

Van Orden, Kimberly A., Tracy K. Witte, Kelly C. Cukrowicz, Scott R. Braithwaite, Edward A. Selby, and Thomas E. Joiner, Jr. 2010. 'The interpersonal theory of suicide', *Psychological Review*, 117: 575-600.

Van Reisen, Mirjam, and Munyaradzi Mawere. 2017. *Human Trafficking and Trauma in the Digital Era: The Ongoing Tragedy of the Trade in Refugees from Eritrea* (Langaa Rpcig: Bamenda, Cameroon).

Vijayakumar, Lakshmi. 2016. 'Suicide Among Refugees – A Mockery of Humanity', *Crisis*, 37: 1-4.

Walker, Jane, Christian Holm Hansen, Isabella Butcher, Neelom Sharma, Lucy Wall, Gordon Murray, and Michael Sharpe. 2011. 'Thoughts of Death and Suicide Reported by Cancer Patients Who Endorsed the “Suicidal Thoughts” Item of the PHQ-9 During Routine Screening for Depression', *Psychosomatics*, 52: 424-27.

Walker, Jane, Rachel A Waters, Gordon Murray, Helen Swanson, Carina J Hibberd, Robert W Rush, Dawn J Storey, Vanessa A Strong, Marie T Fallon, and Lucy R Wall. 2008. 'Better off dead: suicidal thoughts in cancer patients', *Journal of Clinical Oncology*, 26: 4725-30.

WHO. 2014. "Preventing Suicide: A Global Imperative." In. Luxembourg.

Yuval, Kim, Anna Aizik-Reebs, Ido Lurie, Dawit Demoz, and Amit Bernstein. 2021. 'A Functional Network Perspective on Posttraumatic Stress in Refugees: Implications for Theory, Classification, Assessment, and Intervention', *Transcultural psychiatry*, 58: 268-82.

Yuval, Kim, Ariel Zvielli, and Amit Bernstein. 2016. 'Attentional Bias Dynamics and Posttraumatic Stress in Survivors of Violent Conflict and Atrocities New Directions in Clinical Psychological Science of Refugee Mental Health', *Clinical Psychological Science*, 5: 64-73.
